# Case report: A case of giant breast skin warts caused by HPV infection

**DOI:** 10.3389/fonc.2024.1422800

**Published:** 2024-08-20

**Authors:** Chaohui Wang, Yuyang Zhao, Zhenhua Sun, Mingjun Li

**Affiliations:** Department of Thyroid and Breast Surgery, Affiliated Hospital of Jiangsu University, Zhenjiang, China

**Keywords:** human papillomavirus (HPV), condyloma acuminatum (CA), Buschke-Lowenstein tumor, squamous cell carcinoma (SCC), skin tumor

## Abstract

GCA, also known as Buschke-Lowenstein tumor, is a rare sexually transmitted disease associated with HPV types 6 and 111. These warts are considered histologically benign, but there is a risk of localized invasion and development of malignancy. This malignant transformation occurs most often in the perianal and vulvar areas, and involvement of other sites is relatively rare2. In this case, we report a rare case of a giant wart originating from breast skin infected with HPV and progressing to cutaneous squamous cell carcinoma.

## Introduction

Giant condyloma acuminatum (GCA), also known as Buschke–Lowenstein tumor, is a rare sexually transmitted disease associated with HPV types 6 and 11 (1). These warts are considered histologically benign, but there is a risk of localized invasion and development of malignancy. This malignant transformation occurs most often in the perianal and vulvar areas, and involvement of other sites is relatively rare (2). In this case, we report a rare case of a giant wart originating from breast skin infected with HPV and progressing to cutaneous squamous cell carcinoma.

## Case report

A 64-year-old male patient presented with a right breast lump that was first noticed 10 years ago and was not taken seriously by the patient at that time. The lump gradually increased, significantly accelerating growth over the past year. The patient denied other breast lumps and skin changes. The patient had no history of significant medical or surgical disease and denied history of unhealthy sexual activity, but had history of poor personal hygiene as a public bathroom worker and had no family history of similar disease.

**Figure 1:**
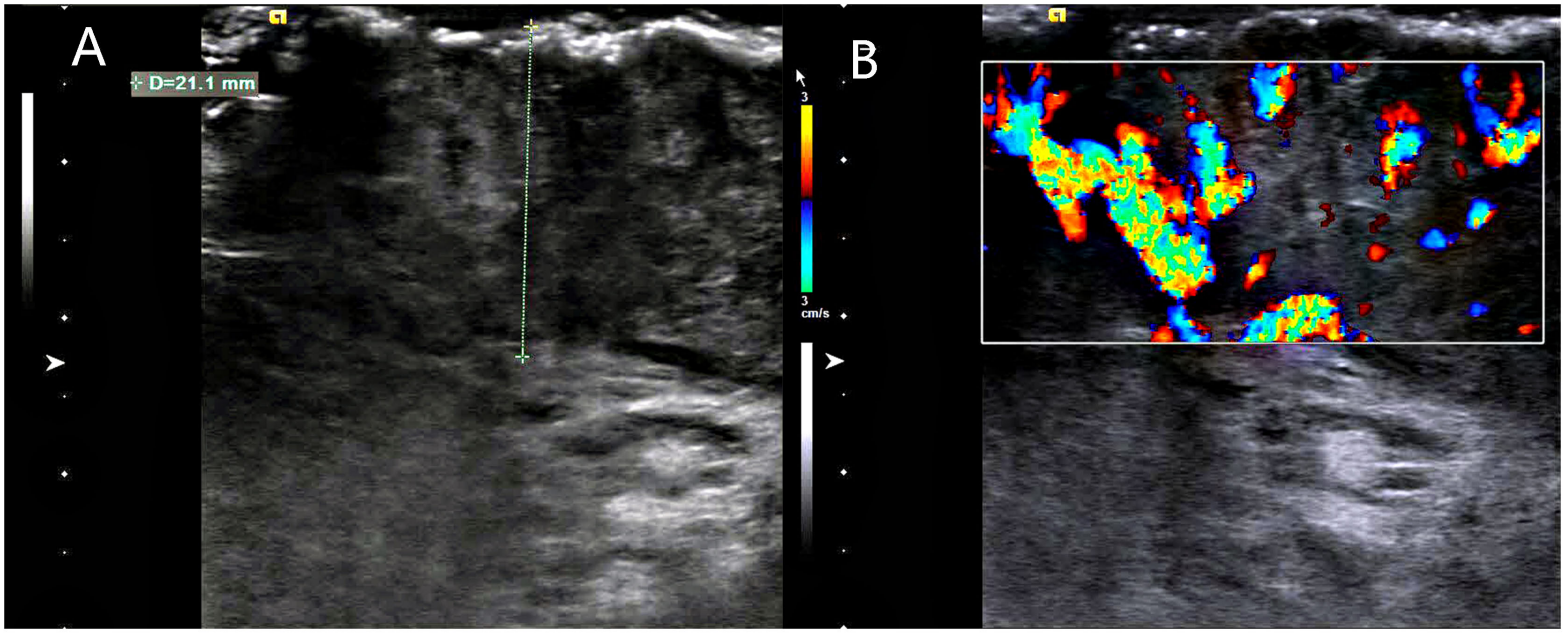
Breast ultrasonography. **(A)** A large mass was seen on the surface of the right chest wall, the thickest part of which was about 21.1mm, and the inner part of the mass was uneven and hypoechoic. **(B)** color Doppler flow imaging(CDFI): abundant blood flow signal is seen inside the mass.

**Figure 2:**
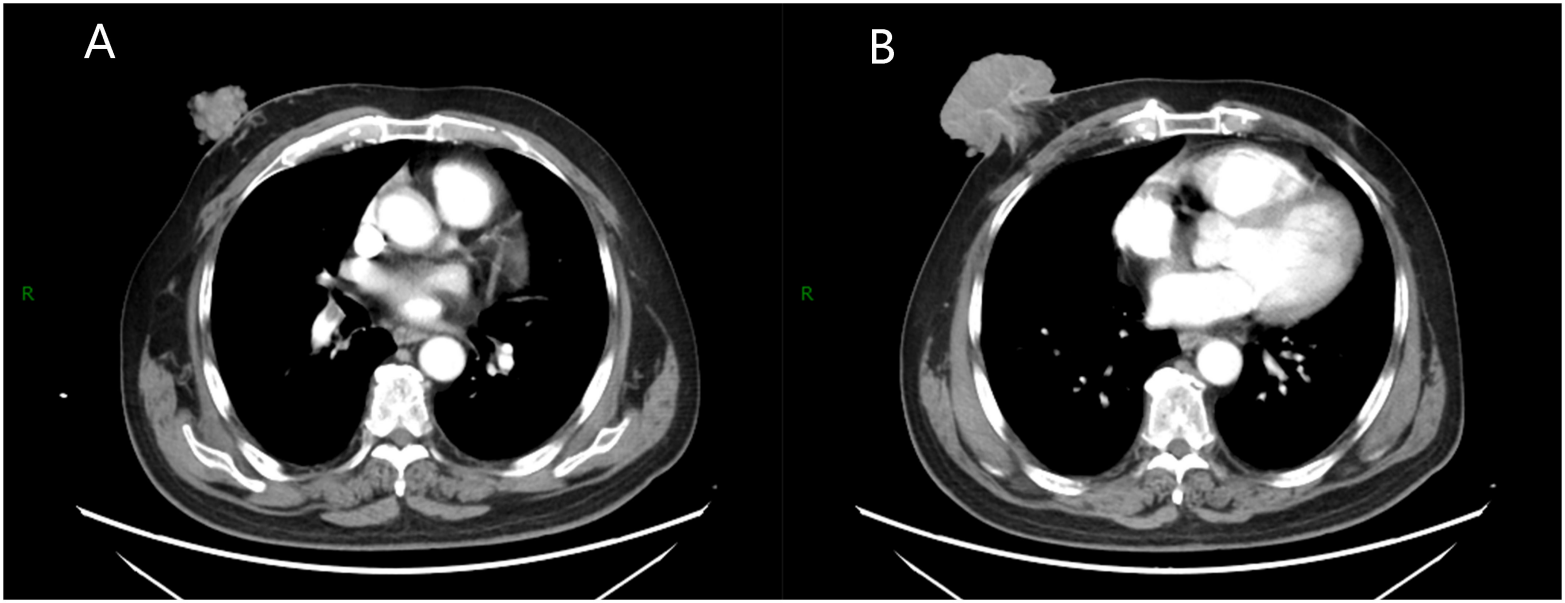
Chest enhanced scan. **(A)** A soft tissue mass with irregular shape and cauliflower-like changes was seen in the right breast area, which was connected with the nipple and showed obvious uneven enhancement. **(B)** The tissue behind the nipple shows thickening and strengthening.

**Figure 3:**
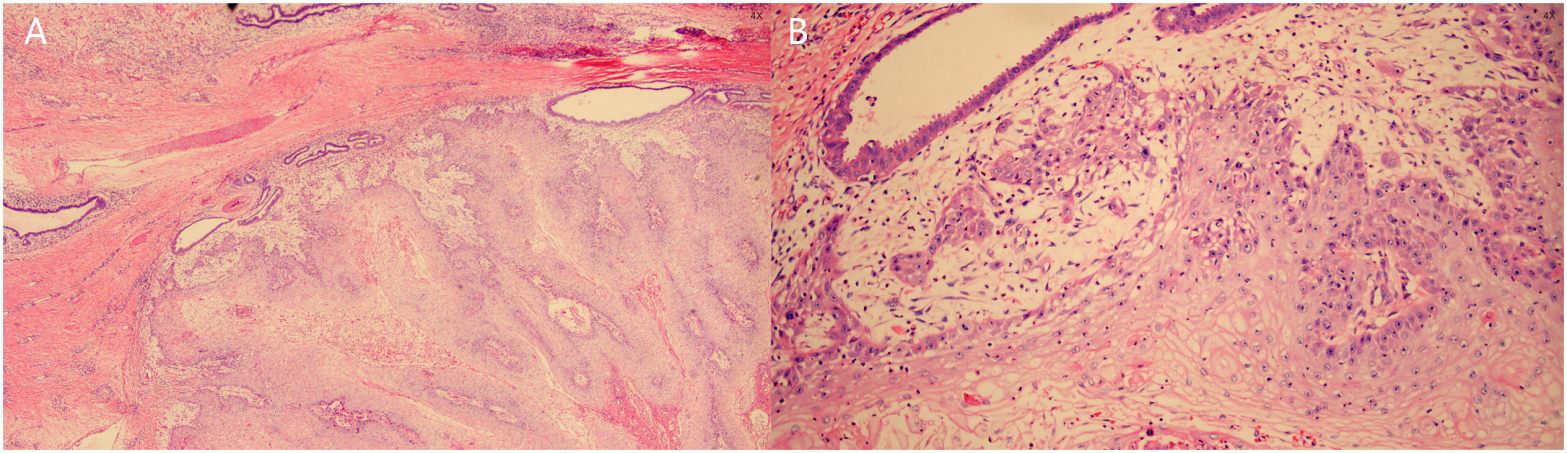
Histopathologic examination. **(A)** Papillary and pestle-and-ball heterogeneous hyperplasia of squamous epithelium with koilocyte formation, chronic suppurative inflammation in some areas, and hyperplasia and dilatation of mammary ducts. **(B)** Squamous epithelial verrucous and papillomatous hyperplasia with some areas of carcinoma, carcinoma areas of highly differentiated squamous cell carcinoma with superficial mesenchymal infiltration of skin and soft tissues.

During breast examination in the supine position, a large, hard, non-pressure mass was found in the patient’s right breast, measuring about 72 mm × 80 mm × 48 mm, with a cauliflower-like appearance and localized ulceration accompanied by purulent secretion. Bilateral breast ultrasonography and computed tomography (CT) of the chest and abdomen were performed. Ultrasonography showed an inhomogeneous hypoechoic mass in the right breast, accompanied by abundant blood flow signals (**Figure 1**). Chest CT examination showed a soft tissue mass of about 72 mm × 80 mm × 47 mm in size in the right breast area, with irregular morphology and cauliflower-like changes, which was connected with the nipple and showed noticeable uneven enhancement. The tissue behind the nipple was also thickened and strengthened (**Figure 2**). The remaining CT examinations of the abdomen and pelvis were negative. Tumor-associated antigen examination showed elevated carcinoembryonic antigen 11.78 ng/mL (standard reference value <5 ng/mL) and squamous cell-associated antigen 6.79 ng/mL (standard reference value <2.5 ng/mL). The patient underwent a lumpectomy under general anesthesia. Intraoperative rapid cytopathological analysis showed abnormal squamous epithelial hyperplasia with scooped cell formation, chronic suppurative inflammation in some areas, and hyperplasia and dilatation of the mammary ducts. We decided that shuttle incision for the right breast be performed to enlarge the resection. Postoperative pathological analysis showed that the lesion tissue was consistent with squamous epithelial warts with squamous cell carcinoma (**Figure 3**). HPV DNA 11 positive was found in the wart specimen. The patient did not receive additional adjuvant therapy after the operation. At regular return visits for 2 years, the patient recovered well and did not see any tumor recurrence.

## Discussion

Condyloma acuminatum (CA) is the most common of the sexually transmitted infections and is caused primarily by HPV infection. A small number of CA show papillary chronic hyperplasia and form giant warts known as Buschke–Lowenstein tumors. Despite the benign appearance of the tumor and the minimal degree of cellular proliferation, the tendency for highly differentiated carcinomas may result in downward compression and displacement of the tumor into deeper tissues rather than direct infiltration or metastasis (3). In 56% of patients with GCA, malignant changes may occur (2). This malignant change may be associated with persistent disease, prolonged chronic inflammatory stimulation, and repeated physical and pharmacologic treatments. In the case we report, it is unclear why this patient developed a large wart on his breast skin. We only know that as a public bathroom worker, this patient is at high risk for HPV infection. Why did the HPV infection lead to the development of a large wart rather than a condyloma acuminatum in the breast skin, and under what factors did the wart progress to SCC of the skin? In this process, host immunity, infected HPV types or variants may be involved in tumor formation and progression (4).

The diagnosis of GCA is mainly based on physical signs and pathological examination. It is necessary to differentiate tumors in the breast area from malignant tumors, and examining blood tumor antigens can help distinguish malignant tumors. CT and ultrasonography are also necessary to determine the involvement of organs and lymph nodes (5), and GCA treatment aims to remove the tumor, not to cure HPV. Although there are no standardized treatment guidelines for GCA, and there is insufficient evidence to show the effectiveness of medical treatments, such as interferon, radiotherapy, and chemotherapy (6), the treatment is based on localized surgical excision, which is ineffective in the treatment of extensive lesions and deep lesions combined with carcinoma and is very difficult to treat (7). The chances of efficacious treatment are limited and could be very tricky. In the case of affecting the whole breast, the psychological impact on the patient has to be considered. Still, the initial imaging suggests that the tumor has invaded the post-nipple tissue. We carefully chose to excise the tumor together with the mammary glands. The postoperative pathological results showed that the tumor did not invade the mammary tissue, and the surgical margins were all negative. For patients who develop SCC malignancy and metastasis, it is essential not to forget subsequent adjuvant therapy.

## Conclusion

We reported GCA originating from the breast skin, demonstrating that breast GCA requiring surgical excision may harbor occult SCC. Pathologic examination is necessary to determine whether malignancy has occurred and whether it has invaded the breast. Follow-up with adjuvant therapy is important for patients who develop malignancy and metastasis of SCC.

## Data Availability

The raw data supporting the conclusions of this article will be made available by the authors, without undue reservation.
